# Training and practice for drug inspectors in China: experiences, challenges, and developments

**DOI:** 10.3389/fmed.2025.1571922

**Published:** 2025-06-04

**Authors:** Jinyao Sun, Lubo Guo, Zhixiang Shi, Fang Wu, Yongze Zhang

**Affiliations:** ^1^Department of Training, School of Continuing Education, China Pharmaceutical University, Nanjing, China; ^2^Department of Pharmacy, Jinan Central Hospital, Jinan, China; ^3^Department of Pharmaceutical English, School of Foreign Languages, China Pharmaceutical University, Nanjing, China; ^4^Department of Business Administration, School of International Pharmaceutical Business, China Pharmaceutical University, Nanjing, China

**Keywords:** China, drug regulation, drug inspector, practice, present and future, training

## Abstract

The professional level and inspection ability of drug inspectors directly determine the quality of inspection and affect drug safety. Drug inspectors need to guarantee that they can effectively perform their duties and better provide reliable supervision by receiving strict qualification authentication, systematic professional training, and standardized behavior management. This paper highlights the current situation of training and practice for drug inspectors in China, particularly the development and achievements in recent years, and examines the current challenges and opportunities facing the drug inspector training system in China and proposes some recommendations for improving the drug inspector training system, aiming to provide some insights for improving the overall quality and modernizing the inspection abilities of drug inspectors in China.

## Introduction

The pharmaceutical industry plays a crucial role in the national economy and people’s livelihood. Its high-quality development relies heavily on solid regulatory support. Professional and specialized inspectors of drugs, medical devices and cosmetics are accredited by the drug regulatory authorities to conduct compliance verification and risk assessment on places and activities of drug research and development, production, and other related activities (1). They constitute an important supporting force for strengthening drug regulation and ensuring drug safety and quality. However, in recent years, drug safety incidents have frequently occurred, posing a serious threat to public health. Additionally, the pharmaceutical industry is undergoing transformation and upgrading, coupled with changes in regulatory policies that have heightened the challenges of drug regulation. As a result, drug inspectors are facing growing challenges in their roles. There is an urgent need to enhance the training system for drug inspectors to ensure that they are well-prepared for drug inspection in the new era (2).

Developed countries and regions such as the United States, the European Union, and Japan attach great importance to drug regulation with particular emphasis on the construction of drug inspection teams. In terms of training implementation institutions, the U. S. Food and Drug Administration (FDA) has established the Office of Training, Education and Development (OTED) to supervise the training of drug inspectors. This includes coordinating training courses and schedules, participant coordination, and effectiveness assessment. The European Union (EU), on the other hand, has established various inspector working groups such as GMDP, GLP, GCP, and PhV to conduct training for drug inspectors across EU member states (3, 4). With regard to tiered and classified training, the U. S. classifies drug inspectors into three levels (I, II and III) based on their knowledge and capabilities. The progression from Level I to Level III generally takes 7 years, with the entire training process being systematic, continuous, and well-planned (5). Japan categorizes drug inspectors into four levels: ordinary inspector, lead inspector, senior inspector, and chief inspector, with clear definitions of the competencies, qualifications, and training required for each level (6, 7). In terms of training content and methods, developed countries have explored and established a diverse training framework. For instance, the FDA employs various training methods such as lectures, laboratory visits, laboratory demonstrations, mock inspections, case studies, seminars, and 483 exercises to enhance inspector training (8). The EU tailors its drug inspector training content based on the development, changes, and existing issues in drug inspection, ensuring both timeliness and practicality (9, 10). Japan has established the Asia Training Center for Pharmaceuticals and Medical Devices Regulatory Affairs (PMDA-ATC) to promote exchange and coordination among drug regulatory agencies and inspectors from different countries (11). In China, the National Medical Products Administration (NMPA) has gradually established a drug inspection team with its own characteristics, which is divided into national and provincial inspectors. National inspectors are primarily responsible for the inspection of drug research and development, drug clinical trials, as well as overseas on-site drug inspection, while provincial inspectors are mainly responsible for on-site inspection of drug manufacturing, distribution, and use. As of the end of 2023, there have been 7,176 drug inspectors with GMP inspection qualifications and 9,076 with GSP inspection qualifications (12).

In this paper, we introduce the current situation of drug inspector training system construction in China from six aspects: training process, training departments, training courses, international training, training methods, and training quality evaluation. We also discuss the challenges and opportunities for drug inspector training system construction in China, with a prospect of the future development and reform direction of the drug inspector training system in the country.

## Training process

**Figure 1:**
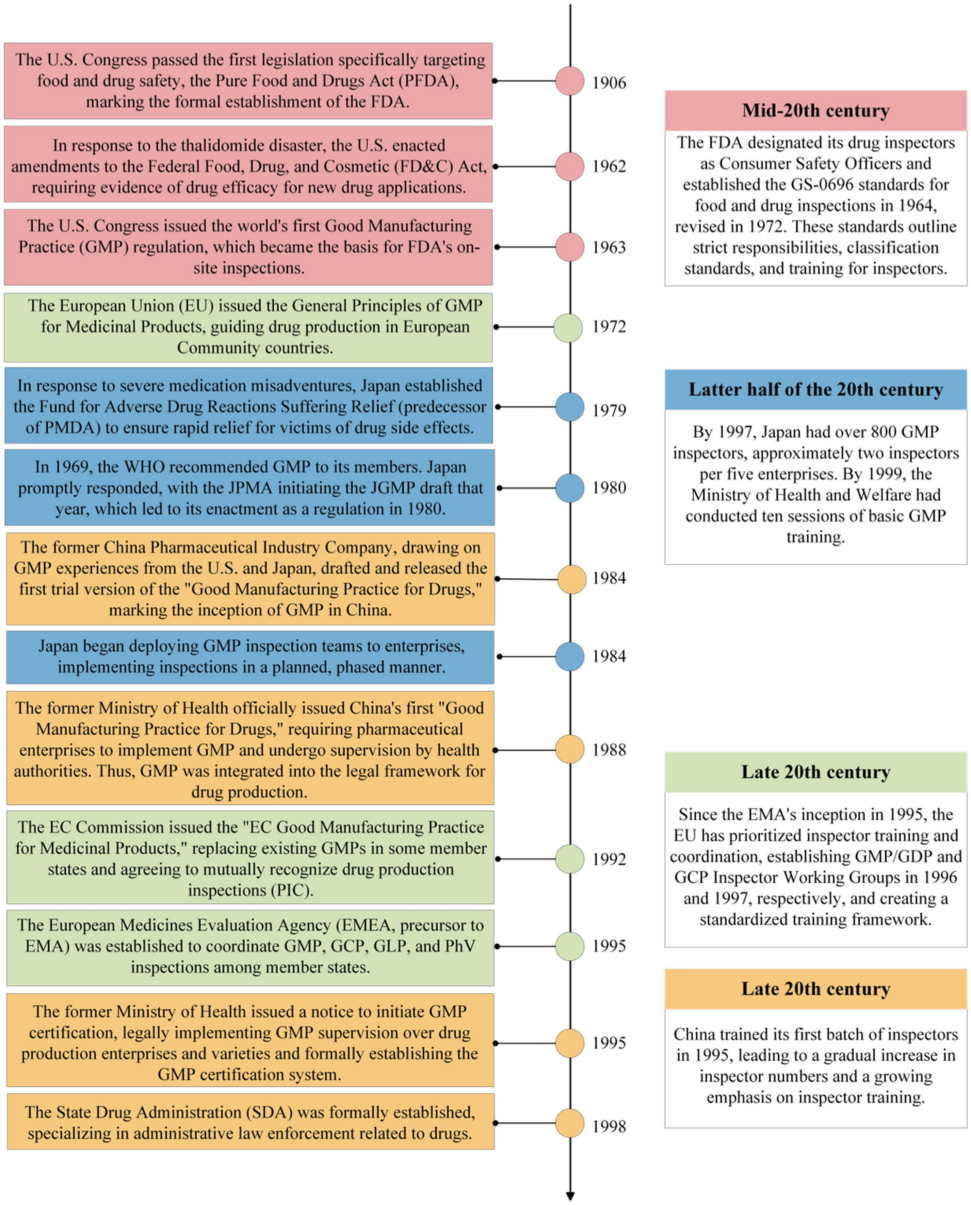
Timeline of drug inspector training in various countries worldwide.

Throughout global history, the establishment and training of drug inspection teams have been closely linked to medication misadventures. These incidents have driven the continuous improvement of pharmaceutical regulations and drug regulatory systems worldwide, leading to higher demands for inspectors’ knowledge and skills, which in turn has prompted the development of specialized training. A significant catalyst for these changes was the “Thalidomide Incident” in the 1960s, which has been regarded as the worst pharmaceutical disaster of the 20th century. This event revealed considerable gaps in drug production and regulation. It encouraged countries to reevaluate their drug safety governance systems, ultimately leading to the creation of stringent drug regulations and regulatory frameworks (13–18). In this context, drug inspection teams to conduct supervision and inspections of drug production and usage have been successively established, ensuring that companies strictly adhere to laws, regulations, and standards (19–22). Simultaneously, training has been employed to enhance drug inspectors’ professional expertise and inspection capabilities. The development and training processes for drug inspectors in the United States, European Union, Japan, and China are illustrated in Figure 1.

**Figure 2:**
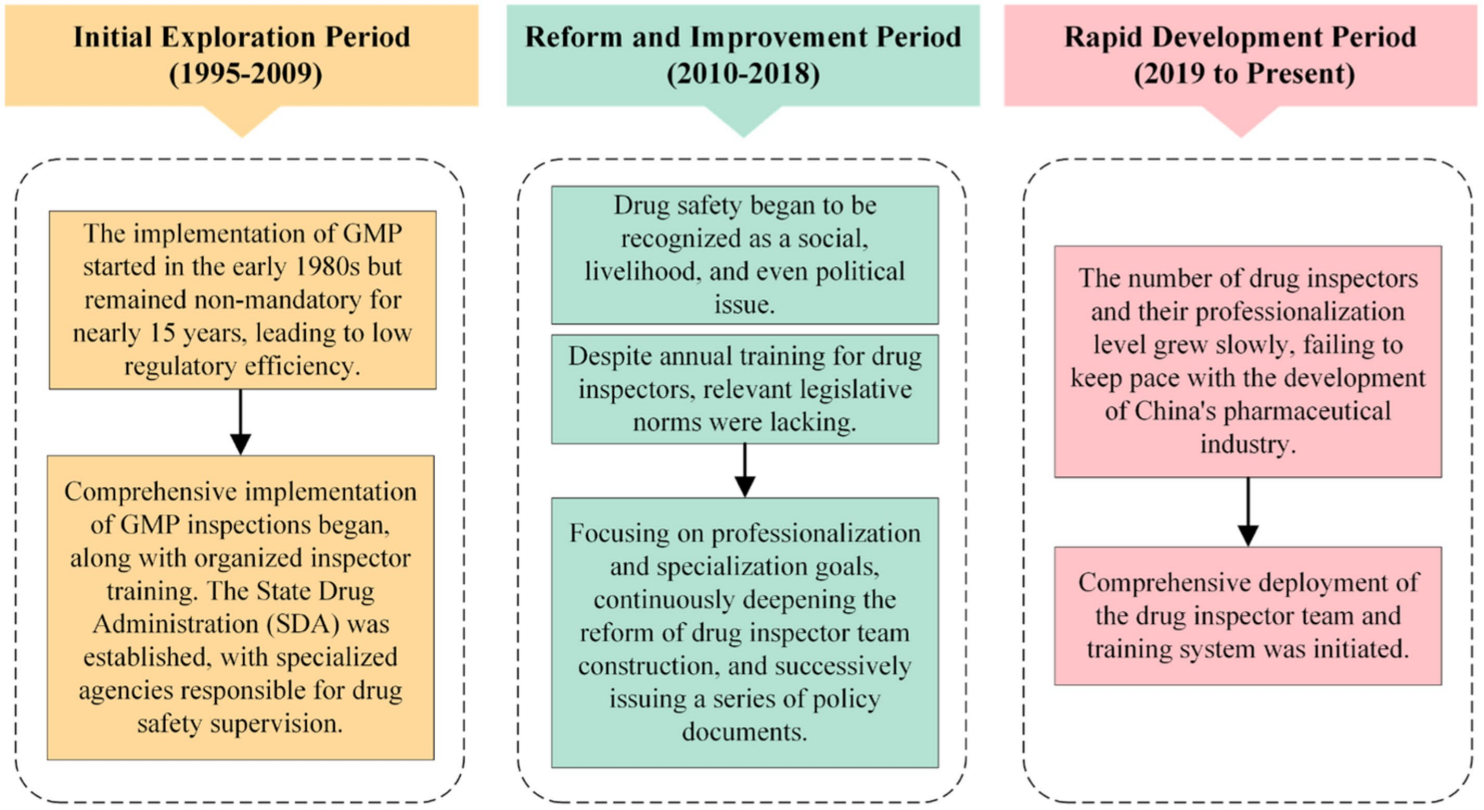
Timeline of drug inspector training in China.

Reviewing the development history of drug inspector training in China reveals that it has undergone three stages: the initial exploration period from 1995 to 2009, the reform and improvement period from 2010 to 2018, and the rapid development period from 2019 to the present (Figure 2). The training of drug inspectors in China began in 1995 when the former Drug Certification Management Center of the Ministry of Public Health trained the first group of GMP inspectors (23). To better carry out GMP compliance inspections for pharmaceutical manufacturers, the second and third groups of GMP inspectors in China were trained and appointed in 1996 and 1998, respectively. The training content included lectures from invited experts on GMP inspection and verification, verification of powder for injection production, and verification of large-volume injection production. By the end of 1998, there had been 166 GMP certification inspectors nationwide (24). In August 1998, the State Council consolidated the State Pharmaceutical Administration (SPA) and the Department of Drug Administration of the Ministry of Public Health, officially establishing the State Drug Administration (SDA), which specialized in administrative law enforcement activities related to pharmaceuticals. Since then, the number of drug inspectors in China has increased gradually. Training for drug inspectors has also gained more and more attention. International inspectors have also been trained through bilateral cooperation, multilateral cooperation, and overseas training (24). The training activities include the 2005 Sino-French GMP Inspection Training Cooperation Project, the 2009 Good Clinical Practice (GCP) Inspection International Symposium, the 2012 GMP International Inspector Training Team, and the 2016 “US Inspector Team Building” lecture.

Since 2019, the construction and training of China’s drug inspection team has entered a stage of vigorous development. In July 2019, the Chinese government issued the “Opinions on Establishing a Team of Professionalized and Specialized Drug Inspectors,” which comprehensively deployed the construction of a professional and specialized drug inspection team, detailing objectives, scale, mechanisms, and training requirements (1). Then, in May 2021, the government issued the “Implementation Opinions on Strengthening the Comprehensive Construction of Drug Supervision Capacity,” which put forward specific requirements for improving the quality of the regulatory team and the provincial drug inspector training program (25). The national drug regulatory authorities also actively explored the construction of the drug inspection team and issued a series of supporting documents such as the “Measures for the Administration of Drug Inspection (Trial Version)” “Measures for the Hierarchical and Classified Management of Professionalized and Specialized Drug Inspectors” “Measures for the Training Management of Professionalized and Specialized Drug Inspectors,” etc. (26–28). The implementation of national policies has facilitated the construction of local drug inspection teams. The provincial drug regulatory authorities have been actively enforcing these requirements and have formulated development plans for the drug inspection team based on actual conditions. Various measures have been taken around training courses, training experts, training methods, training bases, etc. As a result, the construction of drug inspection teams in China has been steadily advancing, and a multi-disciplinary drug inspection team that can meet the regulatory needs has been established. The training system for drug inspectors in China is progressing toward institutionalization and standardization.

## Training departments

**Figure 3:**
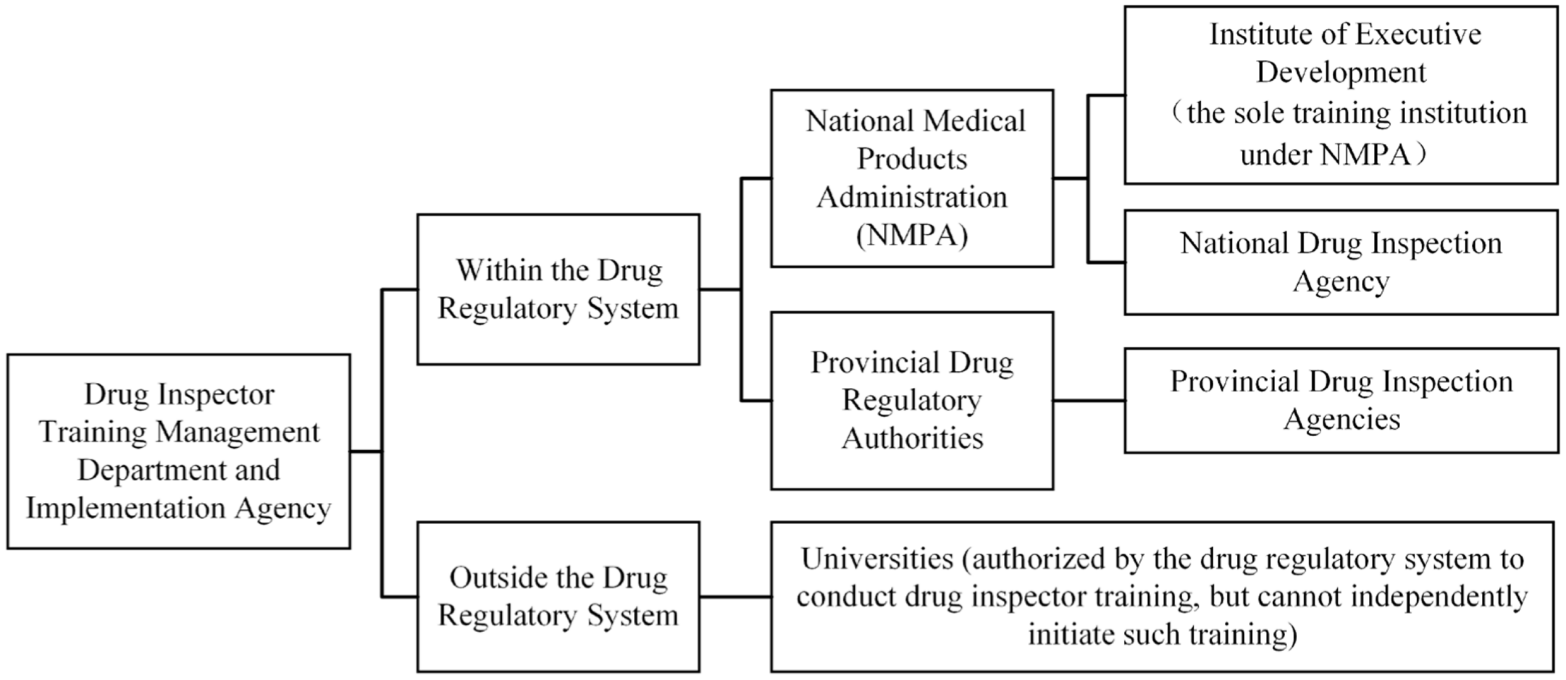
Organizational structure of the drug inspector training department.

In China, the training of drug inspectors is the responsibility of the national and provincial drug regulatory authorities and their affiliated public institutions. At the national level, the NMPA presides over the supervision and administration of drugs throughout the country. As the sole training institution under NMPA, the Institute of Executive Development is responsible for implementing training, which includes training for different levels and types of drug regulatory personnel, such as national inspectors, provincial inspectors, and junior inspectors (29). Thirteen departments under the Institute of Executive Development provide support for various aspects of training, including the Department of Training, the Department of Textbook, the Department of Online Training, and the Department of Development Research. In addition, the Center for Food and Drug Inspection of NMPA (CFDI), as a national drug inspection agency, has set up a Department of Quality Management to take the responsibility for selecting, appointing, training, evaluating, deploying, using, and supervising the performance of national inspectors (30). At the local level, inspectors’ training and evaluation are primarily organized by provincial drug regulatory authorities or their directly affiliated inspection agencies (31). As of 2023, 27 provinces, autonomous regions, and municipalities directly under the central government in China have established drug inspection agencies (32). Some provincial drug regulatory departments utilize high-quality educational resources and teaching experience from universities by commissioning them to design training courses for some drug inspectors or specifically undertake inspector training. The organizational structure of the drug inspector training department is shown in Figure 3.

## Training courses

In China, the training courses organized by the NMPA Institute of Executive Development have the following four characteristics. Firstly, the course system consists of five modules: drug module, medical device module, cosmetics module, emergency drill module, and comprehensive module (33). The drug, medical device, and cosmetics modules mainly focus on professional knowledge and inspection skills training. The emergency drill and comprehensive modules focus on cultivating comprehensive abilities. Secondly, the course type covers the drug life cycle inspection, including drug manufacturing, drug supply, drug clinical trials, pharmacovigilance, traditional Chinese medicine, and vaccine inspection. The course design adopts a combination of cyclical and dynamic training modes. Regular courses are offered regularly, with a cycle of 1–2 months, to reinforce professional fundamentals. In addition, specialized courses are introduced flexibly in response to new regulations, industry frontiers, and regulatory hot topics. The specific course contents are presented in Table 1. Thirdly, the course content covers various aspects such as inspection techniques, inspection standards, regulatory requirements, case analysis, deficiency analysis, and international inspections (Table 2). Fourthly, some training courses emphasize the progressive deepening of knowledge, ensuring that participants build upon their existing understanding and gradually advance to more complex concepts and skills. In 2024, the NMPA Institute of Executive Development designed and developed different levels of online training courses for GMP inspectors, including those for junior inspectors, intermediate inspectors, and senior inspectors (34).

**Table 1:** Some training topics for drug inspectors conducted by the NMPA Institute of Executive Development.

Training frequency	Training types	Training topics	Training time
Regular training	Pharmacovigilance inspection	2024 second drug lifecycle risk management special training course	2024.09.04
		2024 second special training course on pharmacovigilance system and quality management system	2024.08.02
		2024 special training course on traditional Chinese medicine pharmacovigilance	2024.04.19
	Drug manufacturing inspection	Second special training course on policy interpretation and quality management for marketing authorization holders (MAHs) Entrusted with Drug Manufacturing	2024.10.21
		The second special training course on control strategies for cross contamination in multi-product production	2024.08.02
		Special training course on co-production and cleaning validation of drugs	2024.05.13
	Drug clinical trial inspection	Advanced training course on new technologies and quality improvement in drug clinical trials	2024.07.09
		2024 special training course on safety assessment and risk management of drug clinical trials	2024.05.31
	Drug supply inspection	2024 training course on pharmaceutical distribution enterprise quality enhancement	2024.06.24
		2024 drug supply supervision and law enforcement practice training course	2024.04.09
Irregular specialized training	Digital regulation	Pharmaceutical informationization and intelligence special training course	2024.07.09
	Vaccine inspection	Advanced training course on vaccine lifecycle management and regulation science	2024.06.20
	Biologics inspection	2024 case study seminar on biologics change management requirements and change research strategies	2024.06.14
	Regulatory training	Training course on the implementation of the “measures for the quality supervision and administration of the distribution and use of medicinal products”	2024.02.01

**Table 2:** Some training courses for drug inspectors conducted by the NMPA institute of executive development.

Training topics	Training courses
Advanced training course on new technologies and quality improvement in drug clinical trials	Overview of the entire drug development process and frontier new technologies, Overall ideas and principles of ICH E6 (R3) revision, Quality by design (QbD) concept and practice, Core elements of Quality Management System (QMS) and its practical application in GCP, etc.
Special training course on co-production and cleaning validation of drugs	Regulatory requirements for co-production and cleaning validation of drugs, Risk assessment and control strategies for co-production, Full lifecycle risk management for cleaning validation, Deficiencies analysis, etc.
Training course on drug supply supervision and law enforcement practice	Interpretation of the Measures for the Quality Supervision and Administration of the Distribution and Use of Medicinal Products, Analysis of online drug sales business forms and illegal cases, Drug case investigation and key points for coordination between administrative and criminal law under the new situation, Supervision, inspection, and case sharing of drug retail enterprises and medical institutions, etc.
Chinese innovative pharmaceutical enterprise exports drug vigilance special online training course	Enterprise exports strategy—drug vigilance considerations, Drug vigilance regulatory requirements during clinical trials and post-marketing by the US FDA and EU EMA, Practical experience sharing of enterprise exports drug vigilance, etc.
Advanced training course on vaccine lifecycle management and regulation science	Regulation science and regulatory requirements for vaccines, Technical points and implementation of the entire lifecycle of vaccines, Case sharing of innovative vaccine research, Practical operation and on-site visits of key technologies in vaccine research, etc.

## International training

With the globalization of the pharmaceutical industry, the mutual recognition of drug inspections has become the focus of drug regulatory authorities in the world. In recent years, China has actively promoted the coordination and mutual recognition of drug inspection with foreign and international agencies. The NMPA completed the pre-application stage for joining the Pharmaceutical Inspection Co-operation Scheme (PIC/S) in 2021 and became an official applicant in 2023 (35, 36). According to the evaluation criteria for the inspection system of drug regulatory agencies in the PIC/S Audit Checklist (37), China continues to improve drug inspection, including the inspector qualification requirements, number of inspectors, and inspector training program.

In the process of promoting the coordination and mutual recognition of drug inspection, China attaches great importance to improving the global vision of drug inspectors, which can help China better carry out overseas drug inspections. In 2019, the Online Academy of NMPA introduced 102 original online training courses for Chinese inspectors to understand the inspection standards and processes of the US FDA (24). These courses cover various topics, including FDA Bioresearch Monitoring (BIMO), the Food and Drug Act, Quality System Inspection Technology, and Good Manufacturing Practices for Facilities and Equipment. The NMPA also provides diverse international seminars and exchange activities for inspectors. For instance, in 2021, CFDI organized a special training for vaccine inspectors (38). WHO vaccine inspection experts were invited to lecture on GMP inspection and audit experience of biological product manufacturers. Additionally, CFDI arranged for drug inspectors to participate in the second PIC/S Control of Cross-Contamination in Shared Facilities (CCCISF) seminar through an online conference (39). In 2024, the NMPA and WHO collaborated to host the second training for production inspectors of the WHO National Regulatory Authority (NRA) for vaccines (40). The training focused on 10 topics, including risk-based inspection strategies, production and inspection of vaccine stock solutions and preparations, and aseptic processing simulation. The course also involved simulated inspections of vaccine manufacturers in groups.

## Training methods

At present, most provinces in China are actively exploring innovative methods for training drug inspectors. They are dedicated to empowering drug inspector training by combining online training with face-to-face theoretical training with practical training. This includes training inspection team leaders, establishing online training platforms, and setting up training bases. With regard to the training of inspection team leaders, the drug regulatory authorities of Hainan, Zhejiang, and Guangdong provinces continue to promote the training of excellent inspection team leaders, who play a pioneering and exemplary role in providing guidance and assistance for other inspectors. For example, Hainan province has successively formulated training programs for GMP and GSP excellent inspection team leaders, aiming to cultivate excellent leaders through strict selection, “one-on-one” training by senior inspectors, and precise development of training plans (41). With regard to the construction of online training platforms, Shaanxi province has established the “Cool College” online training platform to integrate training resources (42). Similarly, Tianjin has launched a training management system for drug and cosmetics inspectors, with various courses such as laws and regulations, professional knowledge, and inspection practices (43). With regard to the construction of training bases, Heilongjiang province has selected 12 pharmaceutical companies. The training content covers such various parts as blood products, injections, active pharmaceutical ingredients, cosmetics, modern drug logistics, and medical devices (44). Hunan province has also established drug inspector training bases and has fully utilized the training base to carry out on-site training, including rotation and inspection of simulate pharmacies, traditional Chinese medicine processing rooms, and traditional Chinese medicine identification rooms (45).

## Training quality evaluation

Training evaluation is crucial to assessing training effectiveness and ensuring training quality. Drug regulatory authorities at all levels in China have initiated the evaluation of drug inspector training programs. Among these, the training evaluation organized by the Institute of Executive Development is systematic and comprehensive. The evaluation indicators cover various aspects, including training content, trainers, training hours, accommodation, catering, training records, organizational management, gains of participants, and overall evaluation scores, which constitute a holistic evaluation system (29). Owing to regional disparities, industrial characteristics, and regulatory priorities, different provinces have different training evaluation standards for drug inspectors. Some provinces place greater emphasis on the evaluation of inspection theory, while others might focus more on the evaluation of inspection practice or pay more attention to the effectiveness of training. Overall, in terms of basic evaluation content and methods, most provinces conduct competency assessments of inspectors after the completion of training. The assessment content primarily revolves around the materials covered, including laws and regulations, inspection theories, and practical inspection skills, to test the inspectors’ acquisition and mastery of both knowledge and skills. The most common assessment methods are online or in-person exams, supplemented by diverse evaluation approaches such as writing training reflections, group discussions, on-site defenses or presentations, and on-site inspections. Some provinces also use questionnaire surveys to evaluate the overall effectiveness of the training. Additionally, some provinces in China have explored and established distinctive assessment methods. For example, Shaanxi province implements differentiated assessments based on the characteristics of the training methods, with self-study, centralized training, and online training being assessed through written exams, while observation training, simulated inspections, and on-site training are evaluated through on-site assessments (42). Zhejiang province, on the other hand, relies on the “Zhejiang Drug Inspection” platform, developing a “Performance Appraisal” application module to promote the digital transformation of training evaluation (46). In terms of institutional development, Gansu province has issued the “Implementation Measures for the Qualification Examination of Drug Inspectors in Gansu Province (Trial Version),” which requires evaluating the basic knowledge, theory, and practice of drug inspection (47). Candidates must pass all the tested contents within one examination year to obtain the corresponding level of inspector qualification certificate.

## The challenges facing drug inspector training in China

Although significant progress in the drug inspector training system has been made in the past 30 years, it is not enough to form a perfect system, with still some deficiencies. The challenges facing the drug inspector training system in China are in the following five aspects.

Currently, the source of funding for drug inspector training in China is predominantly singular, primarily relying on financial appropriations. Over half of the provinces in China have not received specialized national financial funds for drug inspector training, with the main source of funding being allocations from provincial regulatory authorities. According to preliminary research findings, there is a significant disparity in the amount of training subsidies across provinces. Some regions maintain annual training budgets at relatively basic levels, while more economically developed areas benefit from funding that is severalfold to dozens of times greater, which severely hampers the balanced development of inspector teams.

China faces a shortage of drug inspectors, with a disproportionately high number of part-time inspectors, leading to instability in the inspector team. In terms of the number of inspectors, the 2018 reform of drug regulatory authorities, which retained only provincial drug regulatory authorities, resulted in some inspectors previously employed at municipal and county levels being merged into departments of market regulation. Such personnel changes, including departures and reassignments to other industries or positions, have weakened the staffing of provincial drug inspection agencies. Regarding the distribution of inspectors, China has more inspectors in drug manufacturing and supply but less in drug research and development and clinical trials, particularly in high-risk areas such as vaccines and blood products. In terms of the composition of the inspector team, drug inspectors in China are divided into full-time and part-time. Part-time inspectors constitute a predominant share of the inspection workforce across provinces, serving as a crucial component of the regulatory framework (48). In contrast, developed countries and regions such as the United States, the European Union, and Japan have established their professionalized inspector teams.

The rapid development of the pharmaceutical industry and the dynamic and complex regulatory environment present inspectors with increasing challenges in terms of inspection difficulty and job risks. For example, compared to the 2008 edition of the “Key Points and Determination Principles for Drug Registration Site Inspection,” the 2021 edition of the “Key Points and Determination Principles for Drug Registration Inspection” has increased the number of inspection points for drug research and development sites, production sites, pharmacological and toxicological studies, and drug clinical trials (49, 50). Additionally, newly revised regulations such as the “Drug Administration Law of the People’s Republic of China” and the “Provisions for the Supervision and Administration of Drug Manufacturing” have clearly defined the responsibilities of drug regulation personnel, specifying administrative penalties for scenarios such as concealing, falsifying, delaying, or omitting reports of drug safety incidents, failing to eliminate significant regional drug safety hazards promptly, and investigate identified violations of drug safety laws thoroughly (51). This requires inspectors to keep abreast of dynamic changes in regulatory policies, maintain knowledge updates, and adapt to increasingly stringent and detailed regulatory requirements, continuously enhancing their inspection and enforcement capabilities. Moreover, inspectors face a conflict between training and work, as the intensity of inspection and the frequency of travel are high. This is particularly true for part-time inspectors, who must balance their primary job responsibilities with inspection tasks, thus with limited time and energy to study and delve into inspection knowledge.

The current training course is fragmented, suffering from such problems as the lack of unified training standards, incomplete training content, and lack of specificity, leading to a gap between the expectations and the final training effectiveness. First, provincial drug regulatory authorities train inspectors based on the inspection work, safety supervision situation, and regulatory changes of the previous year (23). The inconsistency in training teachers, course materials, and methods across different provinces leads to regional differences in the professional levels of inspectors and inspection quality. Second, the training course does not fully cover the core competencies required for drug inspectors, with a lack of skills such as stress resilience, adaptability, communication, and teamwork. Third, drug inspectors in China have diverse educational backgrounds, covering disciplines such as pharmaceutical science, biology, chemistry, and law. The differences in educational backgrounds and inspection experience result in different training needs. Although the NMPA has clearly defined inspectors’ hierarchical and classified management in the “Measures for the Hierarchical and Classified Management of Professionalized and Specialized Drug Inspectors,” specific implementation guidelines for hierarchical and classified training have not been issued.

The evaluation of drug inspector training primarily focuses on the training process and immediate learning outcomes, lacking an assessment of the inspectors’ ability to put training into practice and its impact on performance. This makes it difficult to evaluate whether the inspectors’ professional competencies have been enhanced. Evaluations of inspectors’ skill and impact on performance are typically conducted sometime after the training has concluded. This allows inspectors sufficient time to integrate the knowledge and skills acquired during training into their work. This evaluation enables a more authentic and practical observation of the fundamental changes brought about by the training on the behavior of drug inspectors and even the drug regulatory agencies.

## Opportunities and future development directions for drug inspector training

In recent years, China has continuously strengthened its drug regulatory capacity building and is committed to building a professional and specialized inspector team competent in drug regulatory requirements. The newly revised “Drug Administration Law of the People’s Republic of China,” and “Vaccine Administration Law of the People’s Republic of China,” have both put forward their corresponding requirements for professional drug inspectors from the perspective of laws and regulations. These are great opportunities to promote the reform of training for drug inspectors. Looking ahead, constructing the drug inspector training system is a systematic and complex task. The following measures can be taken to improve the drug inspector training system in China.

The training of drug inspectors should focus on enhancing their professional skills, strengthening drug regulatory effectiveness, and ensuring the safety of public medications. It is essential to establish a robust feedback and evaluation mechanism so as to achieve the training objectives. After the training, it is important to promptly assess the drug inspectors’ knowledge acquisition and evaluate the training program. Knowledge acquisition can be measured through exams, inspection reports, mentor evaluations, and on-site inspections. The training evaluation should include surveys and interviews to gather comprehensive feedback from inspectors. Within 6 months to a year, it is necessary to assess how well the inspectors have transferred their knowledge, specifically their ability to apply what they have learned in the training effectively during actual inspections. The evaluation should focus on whether there has been a positive change in the inspectors’ work attitudes and a measurable improvement in their inspection skills. This can be assessed using indicators such as the detection rate of drug quality issues, accuracy of the inspection reports, and the completion of inspection plans. These evaluation methods primarily utilize performance appraisals, interviews, and observation.

Drug inspection is characterized by its high professionalism, independence, technicality, and regulatory nature (52). It is essential to design a hierarchical and categorized course framework, establish a step-by-step training pathway to continuously enhance core competencies among drug inspectors, and promote the integration of training with professional and ranking growth. The following framework should be adhered to: First, with differentiated courses as the main part, the course should be designed around the regulatory needs of the entire drug lifecycle, developing training courses for different types of drug inspectors at various inspection stages. This approach aims to achieve specialization in inspections and precision in training. Second, based on the main part of the course, a progressive course system should be established, encompassing primary, intermediate, advanced, and expert levels. Finally, the structural framework of inspector competency has become more multidimensional, evolving from the singular requirement of “person-job” fit to a diverse and comprehensive requirement encompassing “person-job-organization-environment” fit (53). Therefore, an interdisciplinary course system should be developed, encompassing general knowledge, professional ethics, laws and regulations, international regulation, trends of the industry, inspection fundamentals, inspection operations, and inspection practices.

The training of drug inspectors in China primarily relies on drug regulatory authorities. Social entities are not authorized to conduct drug inspector training independently. Therefore, it is necessary to promote strong partnerships between drug regulatory authorities at all levels and pharmaceutical associations, enterprises, universities, and research institutions, and use high-quality external resources to carry out drug inspector training. For example, drug regulatory authorities can collaborate with universities to develop training courses for drug inspectors and delegate more universities, especially those specializing in pharmaceutical sciences, to conduct drug inspectors training. These institutions already possess mature systems for cultivating pharmaceutical professionals, which enables them to integrate academic courses with professional training programs. In the practical training, drug regulatory authorities can establish a rigorous selection process to identify pharmaceutical companies with comprehensive quality management systems, substantial scale, and good effects as exemplary models in the industry. These companies can be developed into training bases for drug inspectors, providing an immersive and practical training platform. This allows inspectors to deepen their understanding of drug regulatory policies and standards through hands-on experience, enhancing their professional capabilities in on-site inspections and risk assessment.

As direct participants in training, drug inspectors should be encouraged to enhance their enthusiasm and initiative through optimized time management, increased financial investment, and innovative incentive mechanisms. First, a balance needs to be found between inspectors’ work and training. On the one hand, more flexible and diverse training methods should be adopted. For instance, leveraging the advantages of online training, a digital learning ecosystem for drug inspectors can be established, developing a series of high-quality online courses to meet inspectors’ needs for fragmented learning. On the other hand, dedicated training periods should be set for inspectors. During the training, no inspection tasks are scheduled so as to avoid encroaching on their rest time and ensure they can fully commit to the training. Second, financial investment in drug inspector training should be increased, with the establishment of a specialized training budget that covers course development, equipment procurement, expert fees, accommodation and meals, training materials, and transportation costs for on-site inspections. Third, linking inspectors’ participation in training and evaluation results to their job appointments and salary adjustments, exploring the establishment of professional qualification certification for drug inspectors, and improving career development pathways can stimulate their intrinsic motivation for continuous learning.

## Conclusion

The training of drug inspectors is crucial for ensuring drug safety and quality. However, current drug inspector training still faces such problems as limited funding, insufficient motivation among inspectors, lack of systematic training courses, and poor training effectiveness. The training should enhance the core competencies of inspectors, focusing on improving their abilities through progressive training, and enable inspectors to put training into practical skills, thereby improving inspection quality and efficiency. In the future, drug regulatory authorities should prioritize the following actions: First, at the national level, it should be made mandatory for drug inspectors to complete a certain number of training credits annually, with a specified minimum annual training duration. And a training database for inspectors should be established, accurately recording information such as training time, methods, hours, and assessment results. Relevant authorities should regularly supervise and inspect the training status of drug inspectors. Second, strict management of inspector position entry should be enforced, comprehensively implementing a system where inspectors must undergo training and pass assessments before assuming their roles. Third, as a unique group, drug inspectors directly determine drug safety and public health. Therefore, it is necessary to classify drug inspectors as a separate professional qualification category, referencing mature professional systems such as those for teachers, lawyers, physicians, and pharmacists. A standardized professional qualification grading and examination system should be established, categorizing inspectors into primary, intermediate, and advanced levels based on their years of inspection, professional abilities, and training assessment results. Each level should correspond to different job responsibilities and salary standards, enhancing the social status and attractiveness of the drug inspector role and promoting the professional and career development of the drug inspection team.
